# Nonlinear relationship between lumbar bone mineral density and high-density lipoprotein cholesterol in patients with type 2 diabetes

**DOI:** 10.3389/fendo.2025.1570841

**Published:** 2025-06-02

**Authors:** Yu-Hua He, Shi-Li Gu, Yang-Yang Chen, Ming-Mei Xue, Su-Juan Liu, Fang-Fang Guo, Chang-Hua Liang

**Affiliations:** Department of Radiology, The First Affiliated Hospital of Xinxiang Medical University, Weihui, Xinxiang, Henan, China

**Keywords:** bone mineral density, high-density lipoprotein cholesterol, T2DM, fasting blood glucose, mediation effect

## Abstract

**Background:**

High-density lipoprotein cholesterol (HDL-C) plays a significant role in regulating bone mineral density (BMD), with this relationship being influenced by insulin resistance. Although previous studies have investigated the relationship between HDL-C and BMD in patients with type 2 diabetes mellitus (T2DM), the impact of fasting blood glucose (FBG) on this association remains unclear. This study analyzes health screening data from T2DM patients to investigate the relationship between lumbar BMD and serum HDL-C, while also examining how FBG mediates this association.

**Methods:**

This retrospective analysis involved T2DM patients who underwent lumbar BMD screening at our hospital from January 2019 to December 2023. Lumbar BMD was measured via quantitative computed tomography. This study mainly aimed to explore the relationship between lumbar BMD and serum HDL-C in T2DM patients and assess the mediating role of FBG. Statistical analyses employed comprehensive methods: univariate analysis to examine initial variable relationships, generalized additive models for non-linear curve fitting, segmented regression for threshold effects, stratified subgroup analysis to explore potential effect modifications, and mediation analysis to explore potential indirect effect mechanisms.

**Results:**

This work revealed that after controlling confounding factors, an independent nonlinear correlation was observed between lumbar BMD and serum HDL-C in T2DM patients. When HDL-C > 1.35 mmol/L, T2DM patients’ lumbar BMD shows a significant positive correlation [β=3.32, 95% confidence interval (CI): 1.43, 4.21, *P*=0.010)]. Subgroup analysis results indicated the consistent relationship between lumbar BMD and serum HDL-C in T2DM patients across gender (male/female), age (≤40 years/>40, ≤60 years/>60 years), and body mass index (<24 kg/m^2^/≥24, <28 kg/m^2^/≥28 kg/m^2^) subgroups (all *P* interaction > 0.05). Analysis of the mediation effect revealed that FBG mediated 5.38% of the association, with an indirect effect of -0.142 (95% CI: -0.327, -0.026, *P*=0.014).

**Conclusion:**

The independent nonlinear, J-shaped association between lumbar BMD and serum HDL-C in T2DM patients was detected in this study, with FBG negatively mediating this relationship. These findings prove the effect of lipid metabolism and glucose dysregulation on bone health and contribute to the development of osteoporosis prevention and treatment strategies for T2DM patients.

## Introduction

The rapid progress of population aging and changes in lifestyle have led to the increased annual prevalence of type 2 diabetes mellitus (T2DM) and osteoporosis (OP), both of which often coexist (1). OP commonly results in the increased bone fragility and fracture risk (2). Currently, OP is affecting approximately 200 million people worldwide, with China being one of the countries with the highest incidence of bone mass reduction and osteoporotic fractures. The rising total costs of the prevention and management of OP pose a considerable burden on patients and strain healthcare budgets (3). Diabetes is a risk factor for OP, with T2DM disrupting bone structure and leading to bone mass loss (4, 5). Compared with nondiabetic individuals, patients with diabetes not only show higher mortality rates after fractures but also a higher likelihood of multiple complications and poorer prognoses (6). Therefore, the assessment of bone health in T2DM patients is important for the prevention of OP-related fractures.

The interaction between lipid and glucose metabolism on bone metabolism has attracted considerable attention (7–10). High-density lipoprotein cholesterol (HDL-C) is a lipoprotein synthesized by the liver and small intestine and represents one of the most important lipids in the blood lipid profile (11). This molecule can transport cholesterol from peripheral tissues to the liver for metabolism and excretion serves as a protective factor against cardiovascular diseases (12). Numerous studies have linked HDL-C and bone mineral density (BMD), revealing inconsistent findings. Xie et al. (13) used data from the National Health and Nutrition Examination Survey and determined a positive relationship between HDL-C and lumbar BMD in adults aged 20–59 years old. However, other studies have reported conflicting findings, with some demonstrating negative (14) or no significant correlation (15) between HDL-C and BMD. A study of 1,158 elderly patients with T2DM in Beijing, China, revealed that HDL-C as a protective factor against OP in men and women (16). Another prospective research involving 126 female T2DM patients demonstrated a positive correlation between HDL-C and BMD and revealed HDL-C as a crucial predictor of bone mass changes (17). However, Wang et al. observed the lack of correlation between the HDL-C levels and OP in a study involving 1,053 postmenopausal women with T2DM (18). Additionally, Yang et al. (19) used Mendelian randomization to s how that the relationship between lipid profiles and bone density is complex, with findings varying across different populations. Such inconsistent findings underscore the complexity of the relationship between HDL-C levels and bone metabolism, particularly in individuals with diabetes. Therefore, this relationship remains uncertain, especially among patients with T2DM. Currently, limited research exists in T2DM populations, and the role of blood glucose in mediating the HDL-C and bone metabolism relationship is still unclear. This study further clarifies the nonlinear relationship between HDL-C levels and lumbar spine bone mineral density, providing, for the first time, detailed evidence that blood glucose mediates this relationship.

Therefore, in this study, three consecutive years of data were collected from adult T2DM patients during a health screening at our hospital. We mainly aimed to explore the relationship between serum HDL-C levels and lumbar spine BMD in adult T2DM patients undergoing health screening and further investigate the facilitating role of fasting blood glucose (FBG) in this linkage.

## Materials and methods

### Participants and inclusion criterion

This work is a retrospective analysis, and thus, the data was sourced from the medical records of adult T2DM patients who underwent health screening at our hospital’s Health Management Center from January 2019 to December 2023. This study was conducted in accordance with the requirements of the first affiliated hospital of Xinxiang Medical University (Approval Code: EC-024-600). Informed consent was waived because all identifying information of the participants were coded, which ensured that the health screening data used cannot be traced back to individual patients.

**Figure 1:**
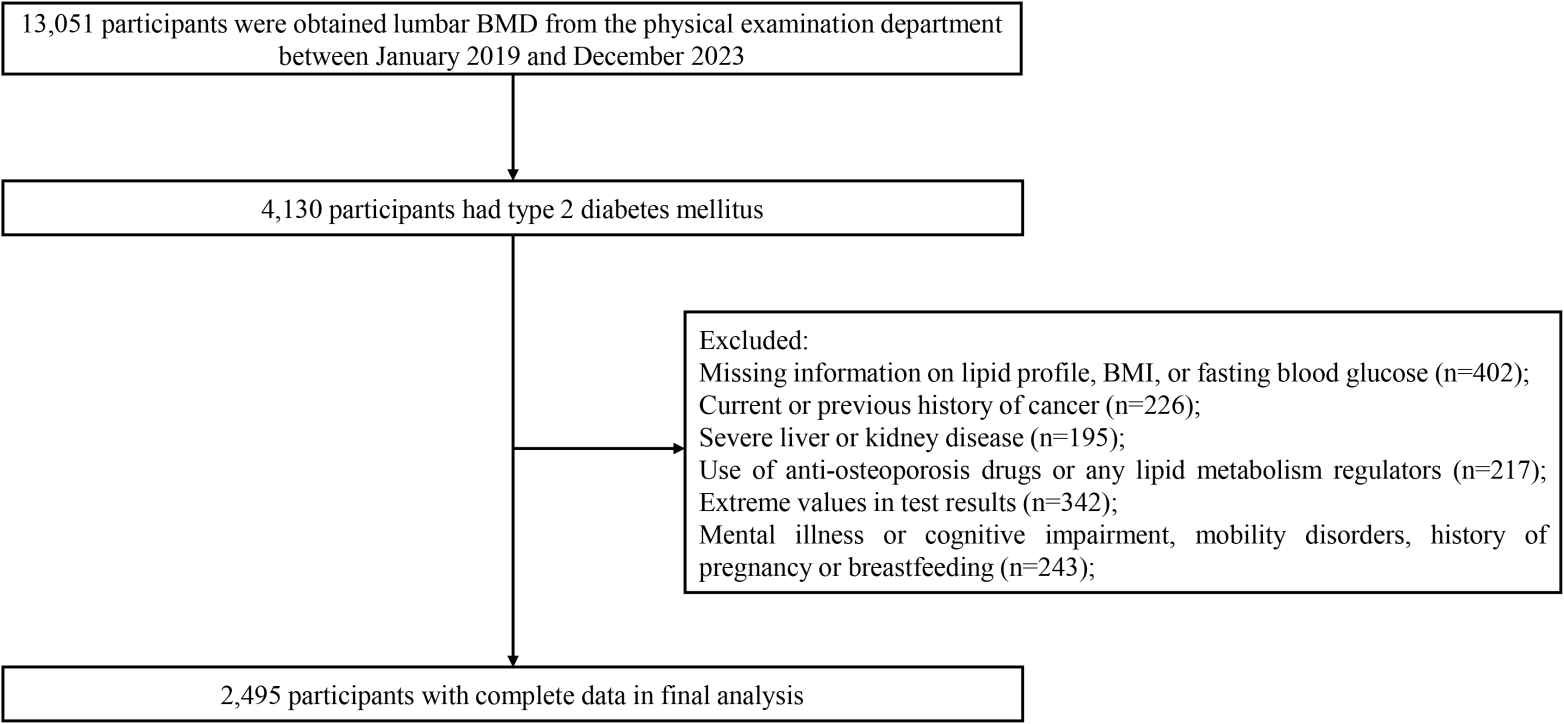
The flow chart of screening T2DM patients.

The inclusion criteria for the study subjects were carefully established to ensure data quality and research validity: (1) confirmed T2DM patients aged between 20 and 80 years, diagnosed according to the American Diabetes Association criteria; (2) participants with comprehensive and verified demographic information; (3) patients who had undergone both BMD and comprehensive lipid profile assessments during their health screening. The exclusion criteria were as follows: (1) past or current cancer diagnosis; (2) history of severe liver or kidney disease; (3) psychiatric disorders, cognitive impairment, mobility impairment, or currently pregnant or breastfeeding; (4) usage of anti-osteoporotic drugs or lipid metabolism regulators; (5) extreme values in test results. The general demographic data of all participants was collected via in-person interviews, which were conducted by trained medical staff and included age, gender, ethnicity, marital status, disease history, and medication history. After multilayer screening, 2,495 T2DM participants, comprising 1,946 males and 549 females, were included. **Figure 1** shows the detailed participant selection process.

### Methods of research

For the scientific integrity and impartiality of data collection, all medical personnel and participants were blinded to the study. Standard training was implemented, and it included all hospital staff involved in data collection. During the screening, all participants completed a questionnaire for basic information collection. This questionnaire was used to record detailed demographic data of the participants. After completion of the questionnaires, the research staff consolidated and organized the data collected. Subsequently, after fasting and abstaining from water for 12 h, measurements of the participants’ height, weight, and blood pressure were performed at 8 a.m. Two measures were implemented for each participant, with the average used as the final data to reduce errors. Body mass index (BMI) was calculated as weight divided by height squared (kg/m²).

T2DM was diagnosed in accordance with the American Diabetes Association criteria (20): a previous physician diagnosis of diabetes or current treatment using hypoglycemic medications, or FBG ≥ 7.0 mmol/L, or glycosylated hemoglobin (HbA1c) level ≥ 6.5%, or 2 h oral glucose tolerance test blood glucose ≥ 11.1 mmol/L, or use of insulin or oral hypoglycemic agents.

### Laboratory measurements

Fasting venous blood was collected from all participants at 8 a.m. The tests comprised the termination of the levels of HDL-C, total cholesterol (TC), low-density lipoprotein cholesterol (LDL-C), triglycerides (TG), total protein (TP), total bilirubin (TB), alanine aminotransferase (ALT), aspartate aminotransferase (AST), gamma-glutamyl transferase (GGT), FBG, HbA1c, creatinine (Cre), and uric acid (UA). An Olympus^®^ AU 5800 automatic biochemical analyzer (Beckman Coulter Inc., Brea, CA, USA) was used in the automatic detection of FBG and blood lipid parameters. Standard laboratory techniques were conducted to evaluate all test parameters.

Systolic blood pressure (SBP) and diastolic blood pressure (DBP) were measured by researchers using an electronic sphygmomanometer (OMRON U30, Omron Corporation, Kyoto, Japan) with the right arm in a semi-flexed position at heart level.

### BMD measurement

Further analysis of the lumbar BMD of all participants was conducted based on low-dose chest computed tomography (CT) during scans to avoid additional radiation exposure. Unified CT scan parameters were used for all participants, and volumetric BMD (vBMD) measurement was performed using Mindways Quantitative Computed Tomography (QCT) Pro 6.1 (Mindways Software, Inc., Austin, TX, USA). The vBMD by QCT is more sensitive to changes in BMD than the areal BMD via dual-energy x-ray absorptiometry (21). Specifically, QCT Pro 6.1 analysis (Mindways Software, Inc., Austin, USA) was used to assess the trabecular vBMD (mg/cm³) of the lumbar spine (L1–L2). Trained professional radiologists measured each vertebra thrice using QCT software and obtained the average of the three measurements as the final BMD for each vertebra. The scientific validity of this method has been confirmed by previously published studies (22).

### Statistical analysis

All statistical analyses were conducted using R version 4.2.0 Foundation (R) and EmpowerStats (http://www.empowerstats.com, X&Y Solutions, Inc., Boston, MA). All statistical tests were two-sided, and P < 0.05 was considered the level of significance.

Normality tests were conducted on all datasets to assess continuous variables. Variables with normal distribution are presented as mean ± standard deviation and skewed continuous variables as median (interquartile range). T-test or rank-sum test was conducted to determine the differences between groups. Categorical variables are presented as frequencies and percentages, and they were compared via chi-square or Fisher’s exact test. The variance inflation factor (VIF) was calculated to detect multicollinearity among model variables, with VIF < 10 indicating zero multicollinearity. Univariate analysis was performed to evaluate the relationship between various variables and lumbar BMD. A generalized additive model (GAM) with a smooth curve fitting was used to explore the nonlinear relationship between lumbar BMD and HDL-C levels after the adjustment for the following variables: sex, age, ethnicity, marital status, BMI, DBP, SBP, TP, TB, ALT, AST, FBG, Cre, and UA. GAM was selected for its ability to flexibly model complex, non-linear relationships without assuming a predefined functional form, which is particularly suitable for capturing potential nuanced interactions between metabolic parameters in diabetes. A two-stage linear regression model was used in the threshold and saturation effect analysis l to determine the inflection point of the correlation between HDL-C and lumbar BMD. Subgroup analysis determined the relationship between lumbar spine BMD and HDL-C levels across various genders, ages, and BMI categories. Finally, mediation effect analysis was performed to determine the role of FBG in facilitating the relationship between lumbar BMD and HDL-C.

## Result

### Baseline characteristics of participants and univariate analysis

This study included 2,495 T2DM participants, and they comprised 1,946 males and 549 females, with an average lumbar BMD of 110.53 mg/cm³ (**Table 1**). Univariate analysis without adjustment for confounding variables revealed age, marital status, SBP, and FBG as risk factors for lumbar BMD in the T2DM participants (all *P*<0.05). Conversely, HDL-C levels, male gender, height, weight, high BMI, DBP, TC, LDL-C, TG, TB, ALT, AST, GGT, and UA served as protective factors for lumbar BMD (all *P*<0.05). Other variables showed no significant correlation (**Table 2** **).**

**Table 1:** Characteristics of the study population.

T2DM (n=2,495)	Statistics
**HDL-C (mmol/L)**	1.24 ± 0.28
Sex, n (%)	
Female	549 (22.00)
Male, n (%)	1,946 (78.00)
Age (year)	
<= 40	73 (2.93)
> 40, <= 60	1,297 (51.98)
> 60	1,125 (45.09)
Nationality n (%)	
Han nationality	2,475 (99.20)
non-Han nationality	20 (0.80)
Marital status, n (%)	
Not married	34 (1.36)
Married	2,461 (98.64)
**High (m)**	1.68 ± 0.08
**Weight (kg)**	73.13 ± 11.16
BMI (kg/m^2^), n (%)	
<24	740 (29.66)
>= 24, < 28	1,218 (48.82)
>= 28	537 (21.52)
**DBP (mmHg)**	78.71 ± 12.66
**SBP (mmHg)**	137.82 ± 20.15
**TC (mmol/L)**	4.79 ± 1.14
**LDL-C (mmol/L)**	2.78 ± 0.90
**TG (mmol/L)**	2.28 ± 1.94
**TP (g/L)**	71.86 ± 4.23
**TB (µmol/L)**	12.78 ± 5.62
**ALT (U/L)**	20.70 (17.10-25.60)
**AST (U/L)**	22.20 (16.50-31.00)
**GGT (U/L)**	28.70 (20.70-44.40)
**FBG (mmol/L)**	7.96 ± 2.32
**HbA1c (%)**	7.36 ± 1.30
**Cre (umol/L)**	70.37 ± 30.13
**UA (umol/L)**	384.52 ± 83.62
**BMD (mg/cm^3^)**	110.53 ± 34.11

HDL-C, high-density lipoprotein cholesterol; BMI, body mass index; DBP, diastolic blood pressure; SBP, systolic blood pressure; TC, total cholesterol; LDL-C, low-density lipoprotein cholesterol; TG, triglycerides; TP, total protein; TB, total bilirubin; ALT, alanine aminotransferase; AST, aspartate aminotransferase; GGT, gamma glutamine transferase; FBG, fasting blood glucose; HbA1c, hemoglobin A1c; Cre, creatinine; UA, uric acid; BMD, bone mineral density; n, number of subjects; %, percentage.

The bolded text represents the variable names or category headers that organize the demographic and analytical data.

**Table 2:** Results of univariate analysis of lumbar BMD.

Variable	Statistics	*β* (95%CI)	*P* value
**HDL-C (mmol/L)**	1.24 ± 0.28	1.96 (0.66, 3.26)	<0.001^***^
Sex, n (%)			
Female	549 (22.00)	Reference	
Male, n (%)	1,946 (78.00)	16.42 (13.26, 19.59)	<0.001^***^
Age (year), n (%)			
≤ 40	73 (2.93)	Reference	
> 40, ≤ 60	1,297 (51.98)	-35.30 (-42.45, -28.14)	<0.001^***^
> 60	1,125 (45.09)	-62.48 (-69.66, -55.30)	<0.001^***^
Nationality n (%)			
Han nationality	2,475 (99.20)	Reference	
non-Han nationality	20 (0.80)	-7.49 (-22.50, 7.52)	0.328
Marital status, n (%)			
Not married	34 (1.36)	Reference	
Married	2,461 (98.64)	-13.35 (-24.89, -1.82)	0.023^*^
**High (m)**	1.68 ± 0.08	87.69 (70.58, 104.79)	<0.001^***^
**Weight (kg)**	73.13 ± 11.16	0.60 (0.48, 0.72)	<0.001^***^
BMI (kg/m^2^), n (%)			
<24	740 (29.66)	Reference	
≥ 24, < 28	1,218 (48.82)	2.22 (-0.88, 5.33)	0.1608
≥ 28	537 (21.52)	7.60 (3.82, 11.38)	<0.001^***^
**DBP (mmHg)**	78.71 ± 12.66	0.40 (0.29, 0.50)	<0.001^***^
**SBP (mmHg)**	137.82 ± 20.15	-0.21 (-0.28, -0.15)	<0.001^***^
**TC (mmol/L)**	4.79 ± 1.14	2.35 (1.18, 3.52)	<0.001^***^
**LDL-C (mmol/L)**	2.78 ± 0.90	2.79 (1.31, 4.28)	<0.001^***^
**TG (mmol/L)**	2.28 ± 1.94	1.75 (1.06, 2.43)	<0.001^***^
**TP (g/L)**	71.86 ± 4.23	0.12 (-0.19, 0.44)	0.441
**TB (µmol/L)**	12.78 ± 5.62	0.36 (0.13, 0.60)	0.003^**^
**ALT (U/L)**	20.70 (17.10-25.60)	0.20 (0.11, 0.28)	<0.001^***^
**AST (U/L)**	22.20 (16.50-31.00)	0.29 (0.23, 0.35)	<0.001^***^
**GGT (U/L)**	28.70 (20.70-44.40)	0.08 (0.05, 0.11)	<0.001^***^
**FBG (mmol/L)**	7.96 ± 2.32	-0.89 (0.31, 1.47)	0.003^**^
**HbA1c (%)**	7.36 ± 1.30	-0.26 (-1.29, 0.77)	0.620
**Cre (umol/L)**	70.37 ± 30.13	-0.00 (-0.05, 0.04)	0.924
**UA (umol/L)**	384.52 ± 83.62	0.04 (0.02, 0.05)	<0.001^***^

HDL-C, high-density lipoprotein cholesterol; BMI, body mass index; DBP, diastolic blood pressure; SBP, systolic blood pressure; TC, total cholesterol; LDL-C, low-density lipoprotein cholesterol; TG, triglycerides; TP, total protein; TB, total bilirubin; ALT, alanine aminotransferase; AST, aspartate aminotransferase; GGT, gamma glutamine transferase; FBG, fasting blood glucose; HbA1c, hemoglobin A1c; Cre, creatinine; UA, uric acid; BMD, bone mineral density; n, number of subjects; %, percentage.

^*^ *P*<0.05, ^**^ *P*<0.01, ^***^ *P*<0.001.

The bolded text represents the variable names or category headers that organize the demographic and analytical data.

### Relationship between HDL-C and lumbar BMD

**Figure 2:**
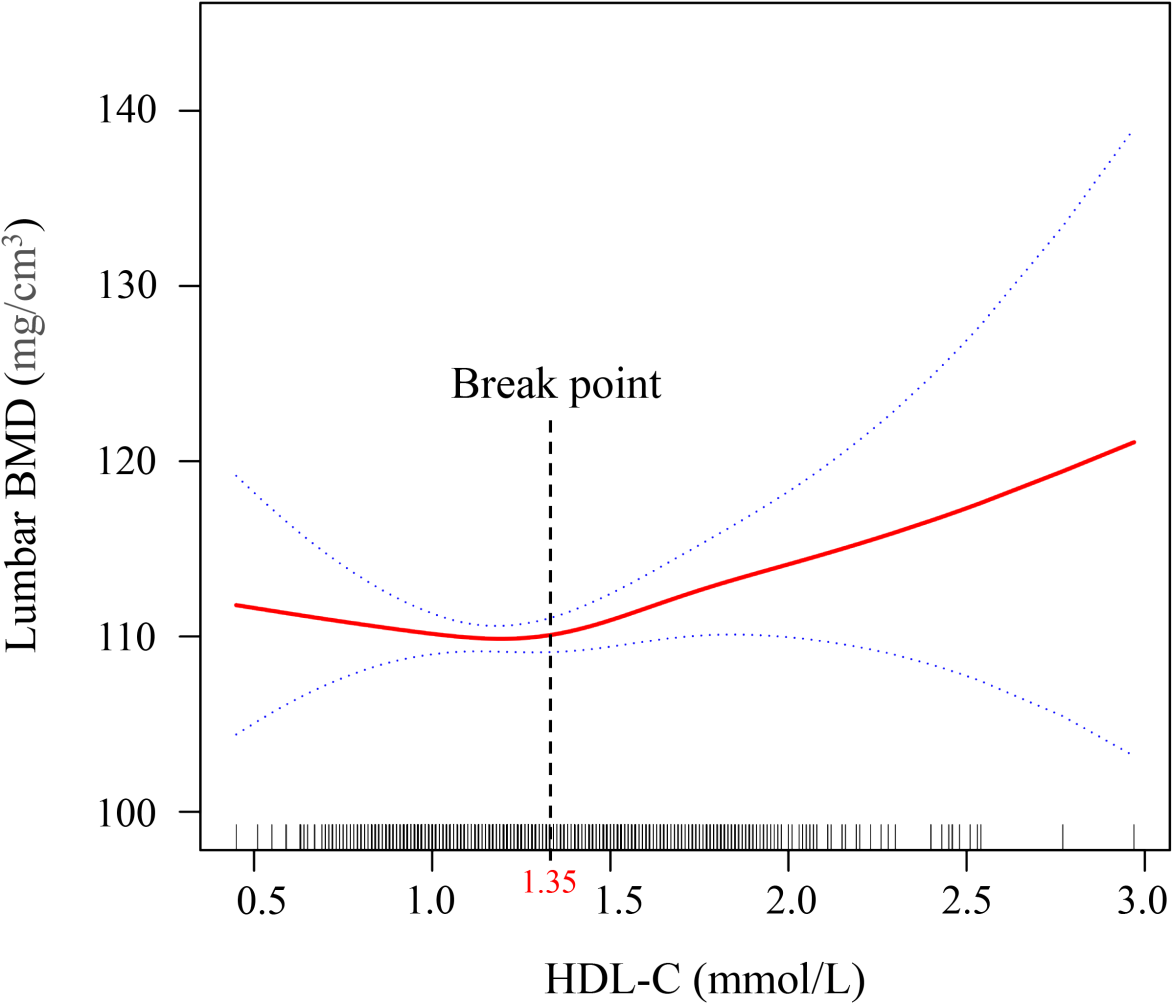
Generalized additive model (GAM) with fitting smoothness for the dose–response relationship between HDL-C and lumber BMD in T2DM patients. The solid red line represents the estimate of lumbar BMD, the dashed blue line represents the confidence interval of the estimate, and the short bottom line represents the sample distribution. BMD, bone mineral density; HDL-C, high-density lipoprotein cholesterol; T2DM, type 2 diabetes mellitus. The threshold was identified using GAM analysis with 3 knots placed at the 10th, 50th, and 90th percentiles of HDL-C, which allows for a flexible, non-linear representation of the relationship between HDL-C and bone mineral density.

Smooth curve fitting using a GAM was employed to explore the non-linear association between lumbar BMD and HDL-C levels in T2DM patients. After adjusting for gender, age, ethnicity, marital status, BMI, DBP, SBP, TP, TB, ALT, AST, FBG, Cre, and UA, a nonlinear J-shaped relationship was observed between lumbar BMD and HDL-C levels (**Figure 2**). We used a two-segment linear model and recursive algorithm and detected.an HDL-C threshold point of 1.35 mmol/L. To the right of the threshold, each unit increase in HDL-C was associated with a 3.32-fold increase in lumbar BMD (95% confidence interval (CI): 1.43–4.21, *P* = 0.010). To the left of the threshold, no significant correlation was detected between lumbar spine BMD and HDL-C levels (*P* = 0.253) (**Figure 2**; **Table 3**).

**Table 3:** The result of the two-piecewise logistic regression model.

Variable	Linear regression	Break point (K)	< K	> K	LLR test
	*β* (95% CI) *P*		*β* (95% CI) *P*	*β* (95% CI) *P*	*P*
**HDL-C**	2.70 (-1.61, 4.01) 0.219	1.35	-2.37 (-3.86, 1.13) 0.253	3.32 (1.43, 4.21) 0.010^*^	0.023^*^

All covariates including sex, age, ethnicity, marital status, BMI, DBP, SBP, TP, TB, ALT, AST, FBG, Cre, and UA were adjusted in this model. CI, confidence interval.

^*^ *P*<0.05.

The bolded text represents the variable names or category headers that organize the demographic and analytical data.

### Subgroup analysis

To comprehensively evaluate the relationship between HDL-C and lumbar BMD, we selected three age groups based on human physiological characteristics. The first group (≤40 years) represents young adults with active bone metabolism; the second group (>40 and ≤60 years) reflects the metabolic transition period of middle-aged individuals; The third group (>60 years) represents the elderly stage with significantly increased osteoporosis risk. This grouping method helps reveal subtle differences in the HDL-C and bone density relationship across different age stages.

**Figure 3:**
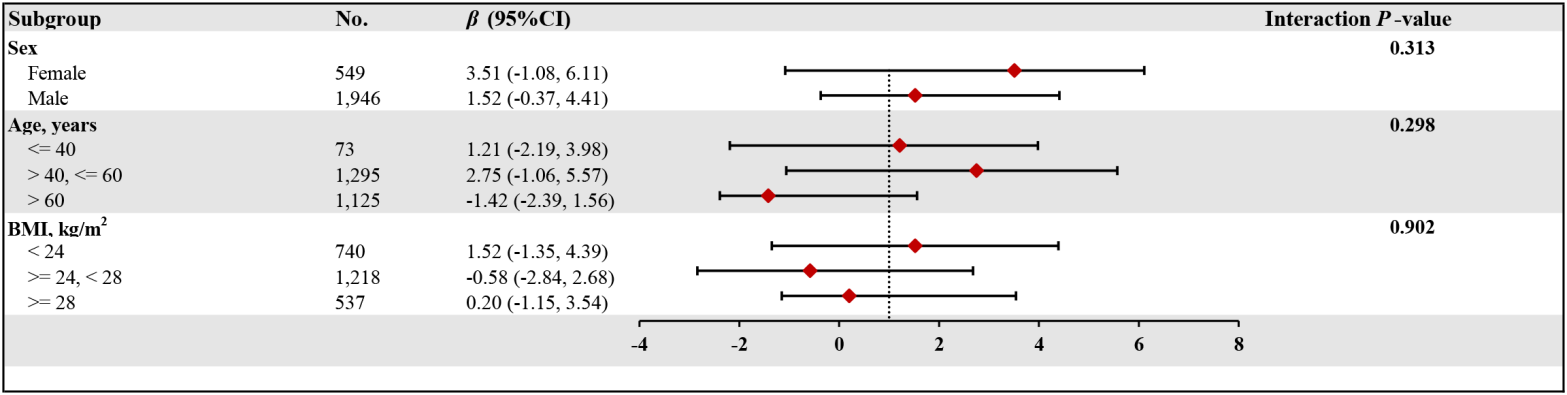
The relationship between HDL-C and lumber BMD of T2DM according to different subgroups. BMD, bone mineral density; HDL-C, high-density lipoprotein cholesterol; T2DM, type 2 diabetes mellitus; BMI, body mass index; CI, confidence interval. Adjusted for all covariates except for this subgroup of variables.

**Figure 3** reveals the subgroup analysis results. The study predefined multiple effect modifiers, including gender (male/female), age (≤40 years/>40, ≤60 years/>60 years), and body mass index (<24 kg/m²/≥24, <28 kg/m²/≥28 kg/m²), to examine the consistency of the association between lumbar BMD and HDL-C levels in T2DM patients. The findings indicate a stable relationship between lumbar BMD and HDL-C levels across different subgroups, with significant physiological implications. This grouping method not only reflects the metabolic characteristics of different populations but also helps reveal the age-, gender-, and body mass-dependent characteristics of the HDL-C and BMD relationship.

### Mediation effect analysis

**Figure 4:**
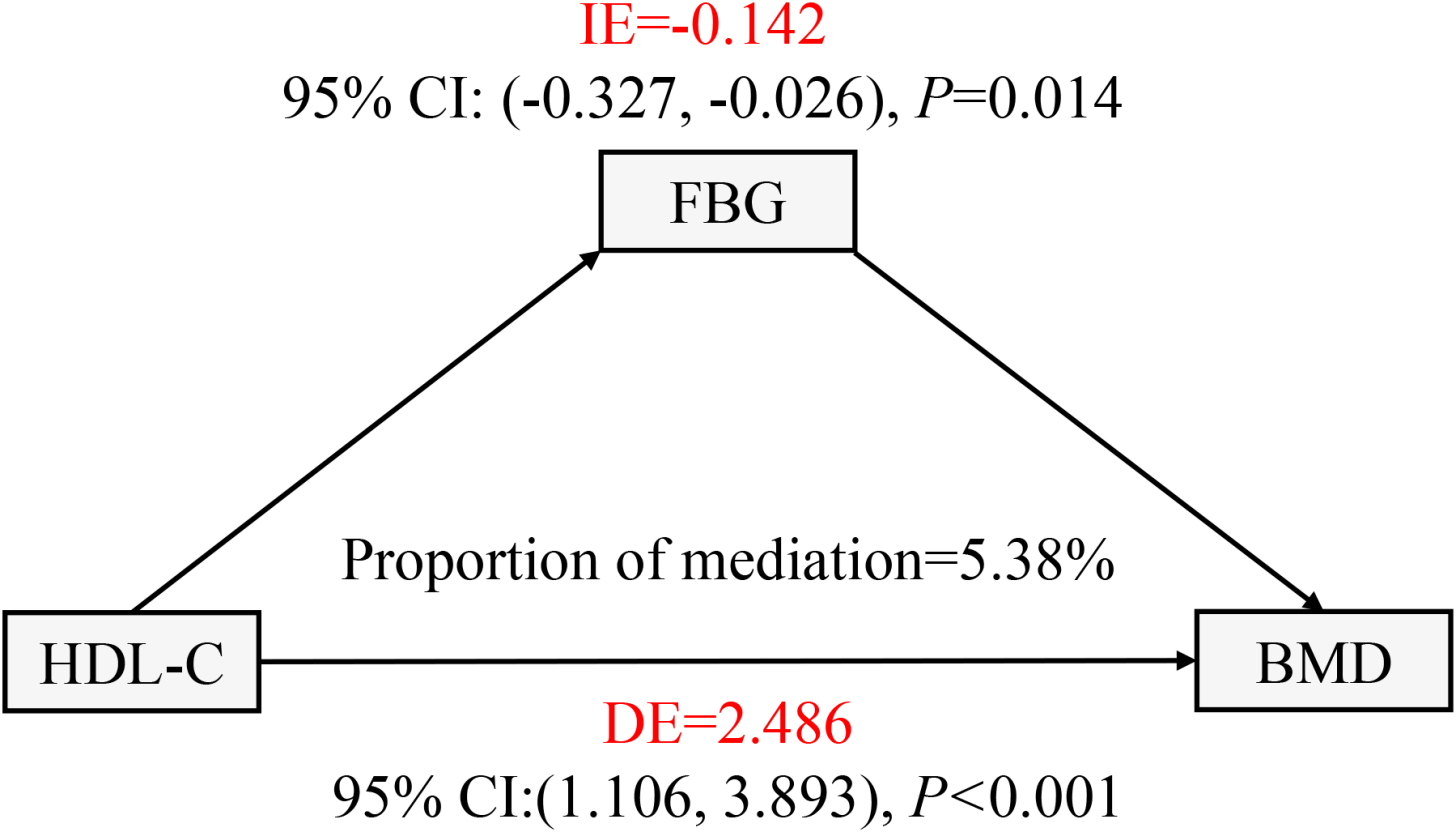
FBG in T2DM patients with the mediation effect of HDL - C relationship with lumbar BMD analysis. FBG, fasting plasma glucose; BMD, bone mineral density; HDL-C, high-density lipoprotein cholesterol; T2DM, type 2 diabetes mellitus; IE, indirect effect; DE, direct effect. All covariates including sex, age, ethnicity, marital status, BMI, DBP, SBP, TP, TB, ALT, AST, Cre, and UA were adjusted in this model.

In this work, mediation analysis was used to explore the mediating role of FBG levels in the relationship between HDL-C and lumbar BMD in T2DM patients. FBG considerably mediated the association between serum HDL-C and lumbar BMD in T2DM patients. In this model, we adjusted for several potential confounders, including sex, age, race, marital status, BMI, DBP, SBP, TP, TB, ALT, AST, Cre, and UA, to ensure the reliability and validity of the results (**Figure 4**). The indirect influence of FBG was -0.142 (95% CI: -0.327 to -0.026, *P* = 0.014), the direct effect of HDL-C levels on lumbar spine BMD reached 2.486 (95% CI: 1.106–3.893, *P* < 0.001), and the mediation proportion was 5.38%.

## Discussion

In the present study, we investigated the relationship between lumbar BMD and serum HDL-C by analyzing three consecutive years of bone density data from T2DM patients at a single Health Management Center. Our comprehensive analysis, after adjusting for potential confounding variables, yielded three key findings. First, we demonstrated a robust and independent nonlinear association between HDL-C levels and lumbar BMD, which persisted across different subgroups of gender, age, and BMI. Second, we identified a critical threshold of 1.35 mmol/L for HDL-C, above which lumbar BMD showed a positive correlation with increasing HDL-C levels. Third, through mediation analysis, we discovered that FBG significantly mediated this relationship, accounting for approximately 5.38% of the total effect. To our knowledge, this is the first study to characterize the nonlinear nature of the HDL-C-BMD relationship in T2DM patients and elucidate the mediating role of FBG. These findings provide novel insights into the complex interplay between glucose and lipid metabolism in bone health, with potential implications for osteoporosis prevention and treatment strategies in T2DM patients.

The interaction between lipid and bone metabolism has been widely explored. The biological mechanism linking lipid and bone metabolisms necessitates the proper differentiation of mesenchymal stem cells (MSCs). During normal functioning of the Wnt/β-catenin signaling pathway, MSCs can further differentiate into osteoblasts, vascular endothelial cells, and adipocytes. However, the disruption of this pathway causes changes in the ratio of osteoblast to adipocyte differentiation (23). Adipocytes in the bone marrow show sensitivity to changes in blood lipid levels, which can increase pressure within the bone marrow cavity and reduce blood flow. This condition potentially leads to the ischemic necrosis of osteocytes and bone loss. In addition, adipokines participate in OP development, with atherosclerosis promoting the release of inflammatory factors that influence bone metabolism and further reduce bone density (24). In T2DM patients, insulin resistance or insufficient secretion leads to enhanced lipolysis in adipose tissue, which results in more free fatty acids penetrating the bloodstream (25, 26). HDL-C, as a key lipid in the body, shows a close relation to various metabolic diseases (27). Prior research suggests a strong link between HDL-C and OP (13). T2DM patients often presented reduced levels of HDL-C (28). However, the relationship between HDL-C levels and lumbar BMD in T2DM patients remains controversial. In this context, investigating the effect of HDL-C on bone density in T2DM patients will provide new evidence for the prevention and treatment of OP in this population and aid clinicians in the development of scientific strategies for lipid management in individuals with T2DM.

This study indicated the positive correlation HDL-C levels with lumbar BMD in T2DM patients before adjusting for confounding variables. After the adjustment of confounding factors, HDL-C levels and lumbar BMD remained independent and nonlinearly correlated. The smooth curve fitting graph reveals that when HDL-C levels exceeded 1.35 mmol/L, the lumbar BMD increased with the rise in HDL-C levels in T2DM patients. This finding demonstrates a complex pattern of consistency and divergence with results from several recent studies. Similarly, Xie et al. (13) observed an association between HDL-C levels and lumbar spine BMD, identifying a U-shaped relationship among males and White populations, with a threshold point at 0.98 mmol/L. Consistent with our findings, their study confirmed a nonlinear relationship between HDL-C levels and bone mineral density. The mechanism underlying this threshold phenomenon may be related to the complex physiological roles of HDL-C in bone metabolism. Huang et al. (29) noted that HDL-C is involved in several biological processes related to bone metabolism, including anti-inflammatory effects, resistance to oxidative stress, and direct interactions with bone cells. The threshold of 1.35 mmol/L observed in our study may represent a critical turning point within these physiological mechanisms. Below this threshold, HDL-C may primarily influence bone metabolism by modulating inflammatory factors within macrophages. Beyond 1.35 mmol/L, HDL-C appears to directly affect bone density by inhibiting bone resorption and promoting bone formation. Nevertheless, it is important to emphasize that the precise physiological mechanisms underlying this phenomenon remain to be fully elucidated. Future research should focus on uncovering the molecular pathways through which HDL-C may impact bone metabolism around this critical threshold. In addition, HDL-C acts a protective factor against bone density reduction in men and women over 60 years with T2DM (16). An observational case-control study involving 243 adult participants unveiled the positive correlation between HDL-C and BMD in female T2DM patients (17). This research provides support to the conclusions of our study. The mechanisms by which HDL-C increases BMD may involve several aspects: HDL-C possesses anti-inflammatory properties, which reduces the production of inflammatory factors during bone resorption and thereby protects bone health (30); HDL-C reduces oxidative stress, which protects osteoblasts (bone-forming cells) and promotes bone formation (31); HDL-C may also promote the maintenance of bone density via the improvement of the lipid environment (32). However, a retrospective study from China involving 525 elderly T2DM patients discovered a negative correlation between HDL-C and BMD (33). This discrepancy may be primarily due to differences in the age and gender of the samples.

Furthermore, our mediation analysis revealed a nuanced and intriguing phenomenon: FBG levels significantly and negatively mediated the relationship between HDL-C levels and lumbar BMD in T2DM patients, accounting for approximately 5.38% of the total effect. Although the mediation effect of FBG was modest at 5.38%, this statistically significant finding unveils critical molecular mechanisms by which hyperglycemia systematically influences bone metabolism. This subtle yet significant pathway highlights the intricate relationship between glucose metabolism and bone health. Notably, this mediating effect exhibited a unique “masking effect” characterized by its opposite direction compared to the total and direct effects, which warrants detailed exploration. This complex relationship can be comprehensively explained through interconnected molecular mechanisms by which hyperglycemia impacts bone metabolism. At the fundamental pathway level, chronic hyperglycemia triggers systemic inflammatory responses and oxidative stress through the activation of NF-κB pathway, which not only disrupts lipid metabolism but also impairs osteocyte function. This cascade leads to increased RANKL expression, enhanced osteoclast genesis, and subsequent bone resorption (14). Mechanistically, while insulin normally promotes bone formation by stimulating osteoblast proliferation and differentiation through the PI3K/Akt signaling pathway, this anabolic effect is significantly weakened in hyperglycemic conditions due to insulin resistance. Similarly, insulin-like growth factor-1 (IGF-1), a crucial regulator of bone metabolism, shows reduced activity under hyperglycemic conditions, resulting in impaired osteoblast function and decreased bone formation (34). The observed “masking effect” suggests that the relationship between HDL-C, FBG, and BMD is more complex than traditional linear models might predict. This nonlinear interaction highlights the intricate metabolic network in T2DM patients, where multiple molecular mechanisms simultaneously and sometimes counterintuitively influence bone metabolism. Another significant mechanism involves the accumulation of advanced glycation end products (AGEs) promoted by hyperglycemia, which bind to their receptors (RAGE) on bone cells. This AGE-RAGE interaction activates multiple downstream signaling pathways, including MAPK and JAK/STAT, leading to increased production of pro-inflammatory cytokines and enhanced osteoclast activity. Moreover, AGEs directly compromise bone quality by forming cross-links with collagen fibers, thereby reducing bone matrix flexibility and increasing fracture susceptibility (35). Interestingly, a recent Mendelian randomization study reported that FBG increases hip BMD while reducing hip bone area, which appears to contradict our findings (34). This apparent discrepancy might be attributed to differences in study populations’ ethnic backgrounds and skeletal sites examined, as bone metabolism can vary significantly across different anatomical locations and ethnic groups. Future research should employ advanced molecular techniques and longitudinal designs to comprehensively elucidate these complex relationships, with a particular focus on understanding the mechanisms underlying the observed “masking effect” in metabolic bone disease. The modest 5.38% mediation effect should not be underestimated. In complex biological systems, even small molecular pathways can signify profound regulatory mechanisms. This finding provides a foundation for future research to explore the nuanced interactions between glucose metabolism, lipid profiles, and bone health.

The findings of this study have significant clinical implications for the prevention and treatment of osteoporosis in patients with T2DM. This research is the first to reveal the mediating role of FBG in the relationship between HDL-C and lumbar BMD, providing actionable and precise insights for clinicians to develop personalized treatment strategies. Our findings reveal HDL-C as a promising potential therapeutic target for osteoporosis prevention in T2DM patients. When HDL-C levels exceed 1.35 mmol/L, a significant positive correlation with lumbar BMD is observed, suggesting a critical threshold for clinical intervention. This discovery highlights the importance of personalized lipid management strategies in preventing bone loss, providing clinicians with a quantifiable and actionable approach to bone health in diabetes patients. This finding advances our understanding by demonstrating the complex interplay between lipid metabolism and bone density and highlighting the importance of individualized patient management. Clinicians should now adopt a more comprehensive approach to T2DM patient care, systematically monitoring HDL-C levels, developing personalized intervention strategies, implementing targeted lifestyle modifications, and considering pharmaceutical interventions when necessary. While the mediating effect of FBG was modest at 5.38%, this finding underscores the intricate molecular mechanisms linking glucose metabolism, lipid profiles, and bone health. We recommend implementing comprehensive intervention strategies in clinical practice: optimizing blood glucose control while simultaneously improving lipid profiles through evidence-based lifestyle modifications and necessary pharmaceutical interventions to prevent osteoporosis. Future research should explore detailed molecular mechanisms, conduct prospective multicenter studies, and develop more sophisticated predictive models for osteoporosis risk in T2DM patients.

## Study strengths and limitations

This study investigated the relationship between HDL-C levels and lumbar BMD in a large sample of T2DM patients, employing rigorous statistical methods, including subgroup analysis, to validate the reliability of results. Additionally, this work was the first to analyze the mediating role of FBG in the relationship between HDL-C levels and lumbar BMD in T2DM patients, providing new insights into the combined influence of glucose and lipid metabolism on bone metabolism. However, several limitations should be acknowledged: First, the cross-sectional design fundamentally limits our ability to determine causal relationships between HDL-C and lumbar BMD. This inherent limitation means we can only establish associations rather than prove direct causation, which necessitates future prospective cohort studies to definitively verify these relationships. Second, despite our comprehensive approach of adjusting for multiple confounding variables, we recognize that objective constraints may have led to overlooking potential influential factors. These unaccounted factors could include, but are not limited to, detailed lifestyle patterns, specific dietary habits, comprehensive medication histories, and individual physiological variations that might significantly impact BMD and lipid metabolism. Third, while our single-center study design provided the advantage of enhanced sample homogeneity, it inherently constrains the broader applicability of our findings. The data collected from a single health management center may not comprehensively reflect the demographic and clinical heterogeneity of the entire T2DM population. Consequently, we acknowledge potential limitations in extrapolating our research conclusions to wider clinical contexts. Given these limitations, we strongly recommend future research adopt multicenter, prospective study designs. Such approaches would not only enhance sample diversity but also enable more robust statistical analyses, potentially providing more generalizable insights into the complex relationship between HDL-C, FBG, and lumbar BMD in patients with T2DM.

## Conclusion

The health screening data from T2DM patients were used as basis to reveal an independent J-shaped relationship between HDL-C levels and lumbar BMD. When HDL-C levels exceeded 1.35 mmol/L, a positive correlation was observed between HDL-C and lumbar BMD. Although the FBG mediation was subtle, it revealed complex interactions between glucose metabolism and bone health. Clinicians should implement targeted interventions to maintain HDL-C levels above 1.35 mmol/L, which represents a critical threshold for improving BMD in T2DM patients. Management strategies should include comprehensive lipid profile optimization, lifestyle modifications, and personalized medical interventions.

## Data Availability

The original contributions presented in the study are included in the article/supplementary material. Further inquiries can be directed to the corresponding author.
